# The Clinical Outcomes, Prognostic Factors and Nomogram Models for Primary Lung Cancer Patients Treated With Stereotactic Body Radiation Therapy

**DOI:** 10.3389/fonc.2022.863502

**Published:** 2022-03-01

**Authors:** Li-Mei Luo, Ying Wang, Pei-Xian Lin, Chuang-Huang Su, Bao-Tian Huang

**Affiliations:** ^1^ Department of Radiation Oncology, Shantou University Medical College, Shantou, China; ^2^ Department of Radiation Oncology, Cancer Hospital of Shantou University Medical College, Shantou, China; ^3^ Department of Nosocomial Infection Management, The Second Affiliated Hospital of Shantou University Medical College, Shantou, China; ^4^ Department of Radiation Oncology, Shantou Central Hospital, Shantou, China

**Keywords:** stereotactic body radiation therapy, primary lung cancer, clinical outcomes, prognostic factors, nomogram model

## Abstract

**Purpose:**

Stereotactic body radiation therapy (SBRT) is a standard treatment for early primary lung cancer patients. However, there are few simple models for predicting the clinical outcomes of these patients. Our study analyzed the clinical outcomes, identified the prognostic factors, and developed prediction nomogram models for these patients.

**Materials and Methods:**

We retrospectively analyzed 114 patients with primary lung cancer treated with SBRT from 2012 to 2020 at our institutions and assessed patient’s clinical outcomes and levels of toxicity. Kaplan–Meier analysis with a log-rank test was used to generate the survival curve. The cut-off values of continuous factors were calculated with the X-tile tool. Potential independent prognostic factors for clinical outcomes were explored using cox regression analysis. Nomograms for clinical outcomes prediction were established with identified factors and assessed by calibration curves.

**Results:**

The median overall survival (OS) was 40.6 months, with 3-year OS, local recurrence free survival (LRFS), distant disease-free survival (DDFS) and progression free survival (PFS) of 56.3%, 61.3%, 72.9% and 35.8%, respectively, with grade 3 or higher toxicity rate of 7%. The cox regression analysis revealed that the clinical stage, immobilization device, and the prescription dose covering 95% of the target area (D95) were independent prognostic factors associated with OS. Moreover, the clinical stage, and immobilization device were independent prognostic factors of LRFS and PFS. The smoking status, hemoglobin (Hb) and immobilization device were significant prognostic factors for DDFS. The nomograms and calibration curves incorporating the above factors indicated good predictive accuracy.

**Conclusions:**

SBRT is effective and safe for primary lung cancer. The prognostic factors associated with OS, LRFS, DDFS and PFS are proposed, and the nomograms we proposed are suitable for clinical outcomes prediction.

## Introduction

Lung cancer is the second most common malignancy and the main cause of cancer-related deaths worldwide (1). Stereotactic body radiation therapy (SBRT) is considered a reasonable, efficient, noninvasive, and safe therapy in primary lung cancer patients who are declining surgery resection or medically inoperable due to comorbid diseases (2–4). SBRT uses image guidance and a precise immobilization system while providing ablation radiation prescription dose to the tumor target and rapidly reducing the dose outside the target to weaken the effects in adjacent critical organs (5). Many retrospective and prospective studies have suggested promising clinical outcomes and safety of lung cancer treated with SBRT, with 3-year overall survival approaching 56% and locoregional control achieving 88% (6). The outcome of SBRT is superior to conventionally fractionated radiotherapy and comparable to surgery for patients who are suitable for surgery (7, 8). However, there are still some failure patterns that have to be considered by the medical community.

It has been widely reported that the clinical factors and dosimetric factors can impact the prognostic of clinical outcomes in lung SBRT patients (9, 10). However, the factors relating to overall survival (OS), local recurrence-free survival (LRFS), distant disease-free survival (DDFS) and progression-free survival (PFS) were not clearly identified until now. On the other hand, apart from the clinical factors, economical and practical dosimetric factors should be fully explored when predicting clinical outcomes (11). Furthermore, patients who undergo personalized treatment can benefit from accurate prediction and more effective post-treatment outcomes (12). Lastly, nomograms derived from multiple factors were rarely combined to evaluate the prognosis in SBRT treatment of lung cancer patients.

The purpose of our present study was to analyze the clinical outcomes and radiation-related toxicity in lung cancer patients receiving SBRT. Furthermore, we aimed to identify clinical and dosimetric factors associated with clinical outcomes and develop the prediction nomogram models to assess clinical prognosis to guide reasonable medical strategies.

## Materials and Methods

### Patients and Treatment Planning

After being approved by our hospital board of ethics, we retrospectively analyzed our database of lung cancer patients from January 2012 to December 2020. Eligible patients with primary lung cancer, declining surgery (or inoperable), informed consent to undergo SBRT were included (N=186). Patients with irradiation sites in the chest wall, mediastinum or thoracic vertebra (N=4), incomplete treatment course (N=1), and metastatic lung cancer (N=67) were excluded. The patients included in the study were immobilized with vacuum bags or using a thermoplastic mask fixation system. We used four-dimensional computed tomography (4DCT) or three-dimensional computed tomography (3DCT) to simulate tumors. The specifications of the CT scanner were as follows: tube voltage 120kvp, tube current 350mA, standard convolution kernel, construction matrix 512 × 512. Then, tumors were delineated following the eclipse treatment planning system (version 10.0 of Varian Medical System, Inc., Palo Alto, CA). In addition, the internal target volume based on 4DCT (ITV4D) were generated combining all the gross tumor volumes (GTVs) contoured at the 10 phases of the respiratory cycle. The internal target volume based on 3DCT (ITV3D) was generated using two methods: (1) the combination of two GTVs of the peak-exhale and peak-inhale respiratory phases to form a single volume; and (2) GTV from observing the motion amplitude under fluoroscopy and reconstructed to form a single volume. A 5 mm margin was added in all directions on the ITV that was defined as the planning target volume (PTV). Besides, we assessed image guidance and target lesion location by cone-beam computed tomography (CBCT) prior to each treatment session.

### Data Collection

Patients’ relevant clinical and dosimetric data were collected at our institution. The clinical data that were collected comprised gender, age, smoking status, Karnofsky performance status (KPS), body mass index (BMI), tumor location, clinical stage, histology, equivalent diameter, GTV, PTV, chemotherapy (or not), hemoglobin (Hb), Neutrophil-to-Lymphocyte ratio (NLR), Platelet-to-Lymphocyte ratio (PLR), immobilization device, and 4DCT (or not). The dosimetric data that were collected considered if the prescription dose covered 95% of the target area (D95), the maximum dose in the whole plan (Dmax), the minimum dose of PTV (PTVmin), mean dose of PTV (PTVmean), the maximum dose of PTV (PTVmax), dose inhomogeneity of PTV (PTVmin/PTVmax), the minimum dose of GTV (GTVmin), mean dose of GTV (GTVmean), the maximum dose of GTV (GTVmax), and dose inhomogeneity of GTV (GTVmin/GTVmax). All the doses were expressed as biologically effective doses (BEDs), which were calculated from the linear-quadratic model, BED = D× [1+D/n(α/β)], D = total dose, n = fraction number, α/β=10. We classified the BMI according to the following categories: underweight (<18.5 kg/m²), normal weight (18.5-25 kg/m²) and overweight (>25 kg/m²). Hemoglobin levels of less than 12 g/dL in women and 13 g/dL in men were diagnosed as anemia.

### Follow up and Endpoints

In-person and/or phone follow-ups were conducted between 4 to 6 weeks after treatment, every three months in one year, and every six months thereafter. We assessed the clinical outcomes during these follow-ups, including OS, LRFS, DDFS, PFS, and toxicities. Tumor local recurrence and distant metastases were identified from biopsy, cytology, or lesion progression on imaging findings. Distant metastases were determined to include both regional metastases and systemic metastases. Toxicity was graded using the RTOG/EORTC criteria (13). Time-to-event outcomes were defined from the first treatment date to the first date of confirmed symptoms.

### Statistical Analysis

Statistical analysis was performed using R Software (version 4.0.2), and the survival curves were estimated using Kaplan-Meier analysis. In addition, the differences between curves were assessed by log-rank test. Pearson’s correlation coefficient and the variance inflation factor (VIF) were evaluated to detect multicollinearity among independent dosimetric factors included in the regression model. We considered a correlation coefficient of > 0.6 between two factors and a VIF of > 10 indicative of multicollinearity (14, 15). The X-tile tool was used to calculate the most suitable cut-off values according to the outcomes for continuous factors (16). Then, univariate and multivariate analyses were performed to identify independent prognostic factors associated with long-term effects using Cox regression analysis. The prediction nomogram models were established with those identified factors to predict OS, LFRS, DDFS, and PFS at the 1-, 2-, and 3-year follow-up. After that, the performance of the prognostic nomograms was assessed by the concordance index (C-index) and calibration curves. The internal validation of the predictive nomogram models was performed using bootstrap analysis. P values were two-sided with values < 0.05 considered statistical significance.

## Results

### Patients’ Characteristics

A total of 114 patients met the eligibility criteria on our database. A summary of patient characteristics is shown in **Table 1**. There were 94 males and 20 females among the final sample group and 89 patients (78.1%) over 60 years old. Most of the primary tumor cases (78.1%) were in peripheral locations. The numbers of patients with adenocarcinoma, squamous cell carcinoma, and unknown pathological conditions were 56 (49.1%), 37 (32.5%), and 21 (18.4%), respectively. A minority of patients (21.1%) were treated with concurrent chemotherapy, and the majority of patients (60.5%) were fixed with thermoplastic masks. Mean tumor volume was 20.95 cm3 (range, 0.10-359.60 cm3), mean equivalent diameter was 3.40 cm, (range, 0.40-8.80 cm). The prescribed dose for SBRT treatment was 15 to 70 Gy total, divided in 1 to 12 fractions, during 1- 3 weeks.

**Table 1:** Lung cancer patients’ clinical and dosimetric characteristics.

Characteristics	Counts (%)/Median (range)
Gender	
Man/Woman	94 (82.5%)/20 (17.5%)
Age[years]	
<60/≧60	25 (21.9%)/89 (78.1%)
Smoking status	
Current/Former/Never	79 (69.3%)/3 (2.6%)/32 (28.1%)
KPS	
<80/≧80	14 (12.3%)/100 (87.7%)
BMI [kg/m2]	
Underweight/Normal weight/Overweight	25 (21.9%)/77 (67.5%)/12 (10.5%)
Clinical stage	
I/II/III/IV	51 (44.7%)/15 (13.2%)/6 (14.0%)/32 (28.1%)
Location	
Central/Peripheral	25 (21.9%)/89 (78.1%)
Histology	
Adenocarcinoma/Squamous cell carcinoma/Unknown	56 (49.1%)/37 (32.5%)/21 (18.4%)
Equivalent diameter [cm]	3.40 (0.40~8.80)
GTV [cm^2^]	20.95 (0.10~359.60)
PTV [cm^2^]	72.55 (6.70~585.30)
Hb [g/L]	
Anemia/Normal	43 (37.7%)/71 (62.3%)
NLR	2.55 (0.05~30.08)
PLR	131.90 (44.33~1125.00)
Chemotherapy	
Yes/No	24 (21.1%)/90 (78.9%)
4DCT	
Yes/No	45 (39.5%)/69 (60.5%)
Immobilization device	
Vacuum bag/Thermoplastic mask	45 (39.5%)/69 (60.5%)
D95 [Gy]	96.00 (22.50~180.00)
Dmax [Gy]	114.33 (25.51~490.87)
PTVmin [Gy]	79.48 (20.19~332.63)
PTVmax [Gy]	114.33 (25.51~490.87)
PTVmean [Gy]	103.69 (23.41~439.73)
GTVmin [Gy]	98.06 (21.94~419.09)
GTVmax [Gy]	113.28 (25.51~466.59)
GTVmean [Gy]	105.92 (23.63~441.19)
PTVmin/PTVmax	0.80 (0.41~0.92)
GTVmin/GTVmax	0.91 (0.70~0.99)

KPS, Karnofsky performance status; BMI, body mass index; PTV, planning target volume; GTV, gross tumor volume; Hb, hemoglobin; NLR, Neutrophil-to-Lymphocyte ratio; PLR, Platelet-to-Lymphocyte ratio; 4DCT, four-dimensional computed tomography; D95, the prescription dose covers 95% of the target area; Dmax, the maximum dose in the whole plan; PTVmin, the minimum dose of PTV; PTVmean, mean dose of PTV; PTVmax, the maximum dose of PTV; PTVmin/PTVmax, dose inhomogeneity of PTV; GTVmin, the minimum dose of GTV; GTVmean, mean dose of GTV; GTVmax, the maximum dose of GTV; GTVmin/GTVmax, dose inhomogeneity of GTV.

### Survival Analysis

**Figure 1:**
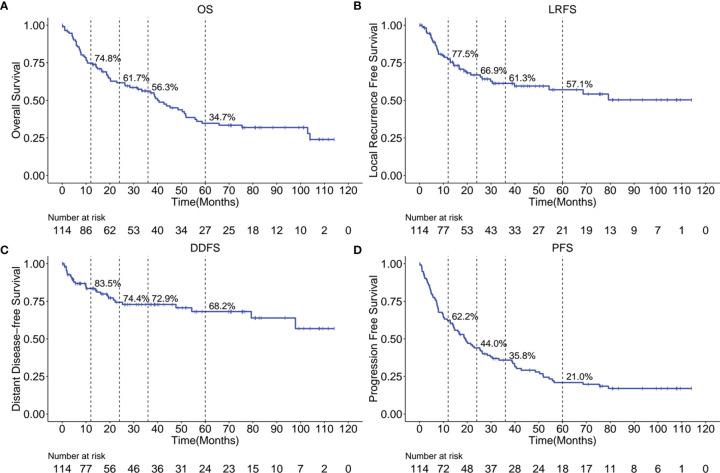
Kaplan-Meier curves showing OS **(A)**, LRFS **(B)**, DDFS **(C)** and PFS **(D)** over time for the entire cohort. OS, overall survival; LRFS, local recurrence free survival; DDFS, distant disease-free survival; PFS, progression free survival.

The Kaplan-Meier OS, LRFS, DDFS, and PFS curves are shown in **Figures 1A–D**, respectively. A total of 67 patients (58.8%) have died, with a median OS of 40.6 months (95% confidence interval (95%CI), 28.4 - 55.7 months). The 1-, 2-, 3- and 5-year OS rates were 74.8%, 61.7%, 56.3% and 34.7%, respectively. We have observed that 40 patients (35.1%) have had a local cancer recurrence, with a median LRFS of 39.8 months. Altogether, LRFS rates at 1-, 2-, 3- and 5- years were 77.5%, 66.9%, 61.3%, and 57.1%, respectively. Furthermore, 29 (25.4%) patients ultimately developed a distant disease for the entire cohort, with a median DDFS of 97.8 months. In this case, the 1-, 2-, 3- and 5-year DDFS rates were 83.5%, 74.4%, 72.9% and 68.2%, respectively. Cumulatively, tumor progression occurred in 85 patients (74.6%), with a median PFS of 14.3 months (95%CI, 19.0 - 28.4 months). We confirmed that PFS rates at 1-, 2-, 3- and 5-year were 62.2%, 44.0%, 35.8%, and 21.0%.

### Continuous Factors Classification

**Figure 2:**
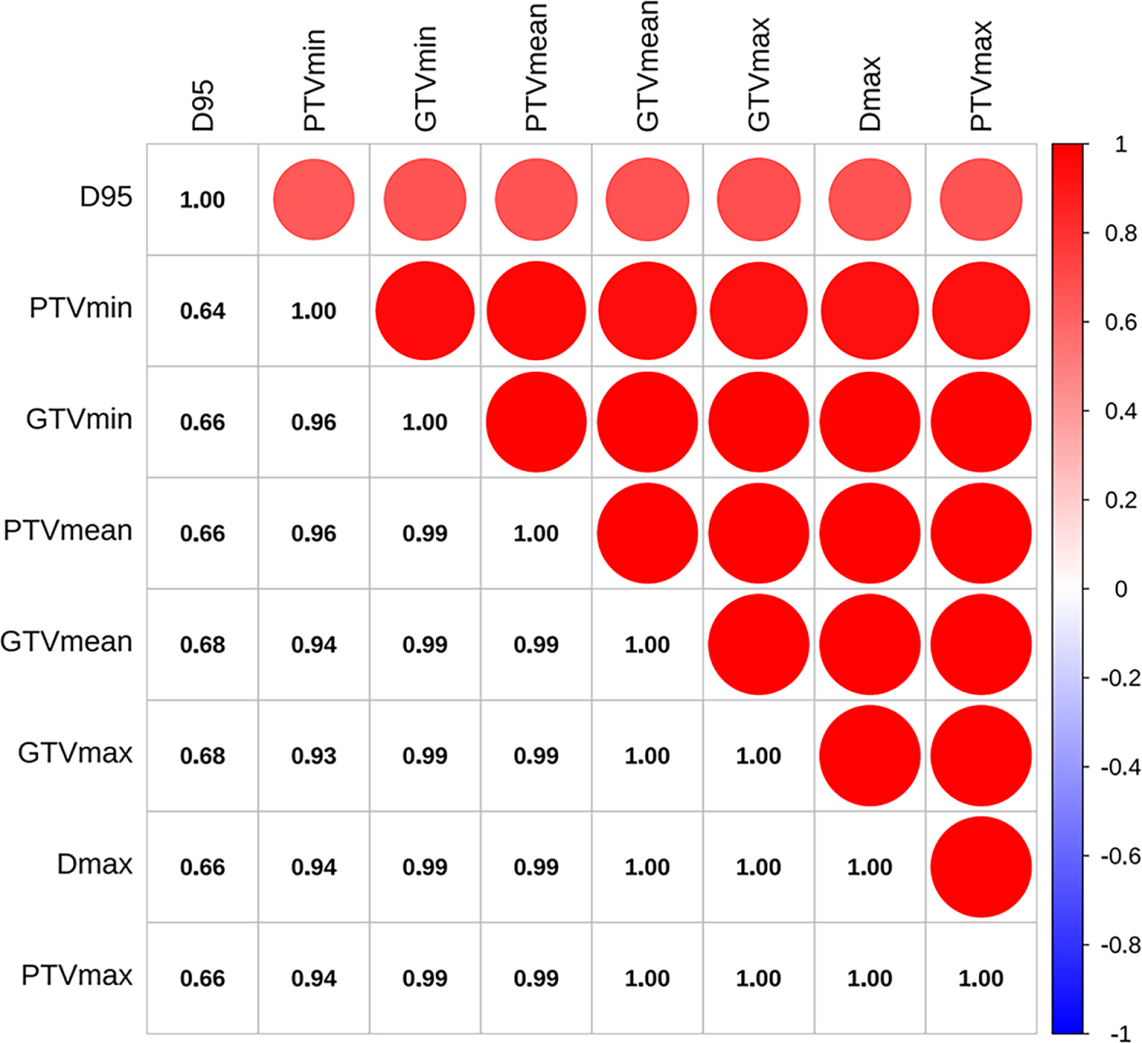
Pearson’s correlation coefficients between pairs of dosimetric factors. D95, the prescription dose covers 95% of the target area; Dmax, the maximum dose in the whole plan; PTVmin, the minimum dose of PTV; PTVmean, mean dose of PTV; PTVmax, the maximum dose of PTV; GTVmin, the minimum dose of GTV; GTVmean, mean dose of GTV; GTVmax, the maximum dose of GTV.

**Figure 3:**
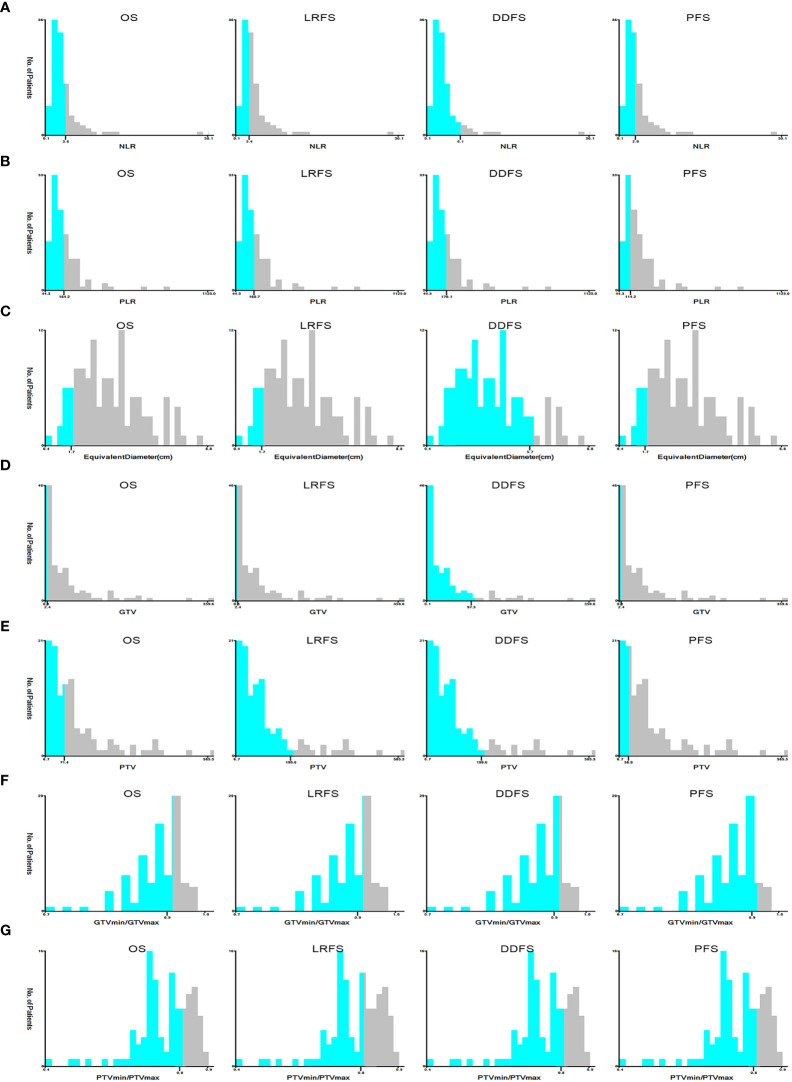
The optimal cut-off values of NLR **(A)**, PLR **(B)**, equivalent diameter **(C)**, GTV **(D)**, PTV **(E)**, PTVmin/PTVmax **(F)**, GTVmin/GTVmax **(G)** in prognosis of OS, LRFS, DDFS and PFS by X-tile software. NLR, Neutrophil-to-Lymphocyte ratio; PLR, Platelet-to-Lymphocyte ratio; GTV, gross tumor volume; PTV, planning target volume; PTVmin/PTVmax, dose inhomogeneity of PTV; GTVmin/GTVmax, dose inhomogeneity of GTV; OS, overall survival; LRFS, local recurrence free survival; DDFS, distant disease-free survival; PFS, progression free survival.

As shown in **Figure 2**, the correlation coefficient among pairs of dosimetric factors was > 0.6, which indicated a moderate or high significant correlation between these factors. As displayed in **Table 2**, except for D95, the VIF values of other dosimetric factors were >10, indicating strong multicollinearity. Therefore, only D95 was included for subsequent analysis and classified by 100Gy. The optimal cut-off values of continuous factors for predicting OS, LRFS, DDFS, and PFS are illustrated in **Figure 3**. As it can be seen, the optimal cutoff values of NLR based on OS, LRFS, DDFS, and PFS were 3.62, 3.40, 6.13, and 2.91, respectively. The PLR cut-off values were 164.17, 160.71, 170.05, and 114.23, and the cut-off values of equivalent diameter were 1.70cm, 1.70cm, 5.70cm, and 1.70cm, respectively. Moreover, the cut-off values of GTV were 2.40cm^2^, 2.40cm^2^, 97.30cm^2^, and 2.40cm^2^ and the PTV cut-off values were 71.40cm^2^, 199.60 cm^2^, 199.60 cm^2^, and 36.90 cm^2^,. The cut-off values of GTVmin/GTVmax were 0.92, 0.92, 0.93, and 0.94, respectively. The PTVmin/PTVmax cut-off values were 0.83, 0.80, 0.83, and 0.83. We classified these factors into two levels according to the above-mentioned values.

**Table 2:** VIF values of dosimetric factors.

	D95	Dmax	PTVmin	PTVmax	PTVmean	GTVmin	GTVmax	GTVmean
VIF value	5.57	8035.82	10.25	9560.41	162.84	53.49	2075.33	415.98

VIF, Variance inflation factor. D95, the prescription dose covers 95% of the target area; Dmax, the maximum dose in the whole plan; PTVmin, the minimum dose of PTV; PTVmean, mean dose of PTV; PTVmax, the maximum dose of PTV; GTVmin, the minimum dose of GTV; GTVmean, mean dose of GTV; GTVmax, the maximum dose of GTV.

### Prognostic Factors Predicting OS, LRFS, DDFS and PFS

**Table 3** outlines the results of univariate analyses, identifying potential prognostic factors associated with long-term outcomes after SBRT. The univariate analysis showed that gender, clinical stage, histology, PTV, PLR, immobilization device, 4DCT (or not), and D95 are associated with OS. At the clinical stage, PTV and immobilization devices were significantly associated with LRFS. It has been observed that the smoking status, clinical stage, Hb, and immobilization device were statistically significant factors for DDFS. In addition, the KPS, clinical stage, histology, PTV, NLR, PLR, immobilization device, and D95 emerged as important potential factors for PFS.

**Table 3:** The univariate analysis of clinical and dosimetric predictive factors for OS, LRFS, DDFS and PFS in lung cancer patients treated with SBRT.

Factors	OS		LRFS		DDFS		PFS	
	HR	P value	HR	P value	HR	P value	HR	P value
Gender (Man vs Woman)	0.505	0.048	1.237	0.563	1.168	0.723	0.911	0.727
Age [years] (<60 vs ≧60)	1.341	0.346	0.538	0.062	0.867	0.744	0.965	0.888
Smoking status (Current vs Former vs Never)	0.862	0.283	1.289	0.120	1.530	0.024	1.105	0.391
KPS (<80 vs ≧80)	0.575	0.108	0.705	0.467	0.438	0.074	0.505	0.025
BMI [kg/m2] (Underweight vs Normal weight vs Overweight)	1.520	0.302	1.745	0.297	2.533	0.207	1.808	0.098
Clinical stage (I vs II vs III vs IV)	1.508	<0.001	1.639	<0.001	1.423	0.017	1.527	<0.001
Location (Central vs Peripheral)	1.045	0.876	1.169	0.681	1.817	0.227	1.012	0.963
Histology (Adenocarcinoma vs Squamous cell carcinoma vs Unknown)	1.568	0.006	1.007	0.975	0.973	0.918	1.341	0.047
Equivalent diameter [cm] (Low value vs High value)	0.567	0.158	0.936	0.912	0.982	0.981	0.676	0.295
GTV [cm^2^] (Low value vs High value)	0.567	0.158	0.936	0.912	0.982	0.981	0.676	0.295
PTV [cm^2^] (Low value vs High value)	1.791	0.020	3.044	0.008	0.764	0.717	1.693	0.037
Hb [g/L] (Anemia vs normal)	0.865	0.569	1.012	0.973	0.474	0.046	0.764	0.227
NLR (Low value vs High value)	1.658	0.052	1.495	0.236	1.953	0.177	1.922	0.003
PLR (Low value vs High value)	1.769	0.032	1.706	0.109	1.530	0.297	1.719	0.019
Chemotherapy (Yes vs No)	1.452	0.278	0.796	0.548	1.000	1.000	0.896	0.682
Immobilization device (Vacuum bag vs Thermoplastic mas)	0.551	0.015	0.418	0.006	0.429	0.024	0.529	0.004
4DCT (Yes vs No)	1.701	0.048	1.241	0.517	1.457	0.352	1.258	0.313
D95 [Gy] (<100 vs ≧100)	0.422	0.001	0.836	0.575	0.459	0.051	0.590	0.018
PTVmin/PTVmax (Low value vs High value)	1.220	0.442	0.841	0.608	1.266	0.576	0.882	0.736
GTVmin/GTVmax (Low value vs High value)	0.915	0.737	0.777	0.435	1.387	0.401	1.075	0.756

KPS, Karnofsky performance status; BMI, body mass index; PTV, planning target volume; GTV, gross tumor volume; Hb, hemoglobin; NLR, Neutrophil-to-Lymphocyte ratio; PLR, Platelet-to-Lymphocyte ratio; 4DCT, four-dimensional computed tomography; D95, the prescription dose covers 95% of the target area; PTVmin/PTVmax, dose inhomogeneity of PTV; GTVmin/GTVmax, dose inhomogeneity of GTV; OS, overall survival; LRFS, local recurrence free survival; DDFS, distant disease-free survival; PFS, progression free survival; HR, hazard ratio.

**Figure 4:**
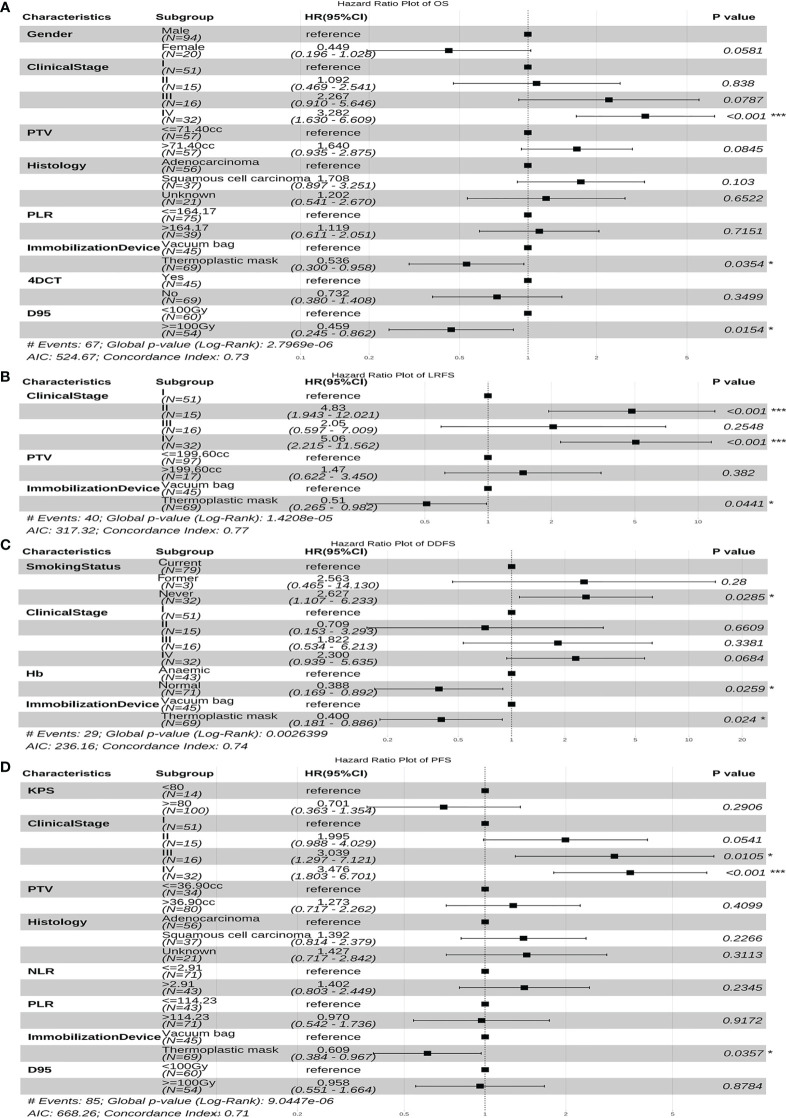
The forest plot of the multivariate cox regression analyses of risk factors for OS **(A)**, LRFS **(B)**, DDFS **(C)** and PFS **(D)**. *p-value < 0.05, ***p-value < 0.001. PTV, planning target volume; Hb, hemoglobin; NLR, Neutrophil-to-Lymphocyte ratio; PLR, Platelet-to-Lymphocyte ratio; 4DCT, four-dimensional computed tomography; D, the prescription dose covers 95% of the target area; OS, overall survival; LRFS, local recurrence free survival; DDFS, distant disease-free survival; PFS, progression free survival; HR, hazard ratio; CI, confidence interval.

**Figure 5:**
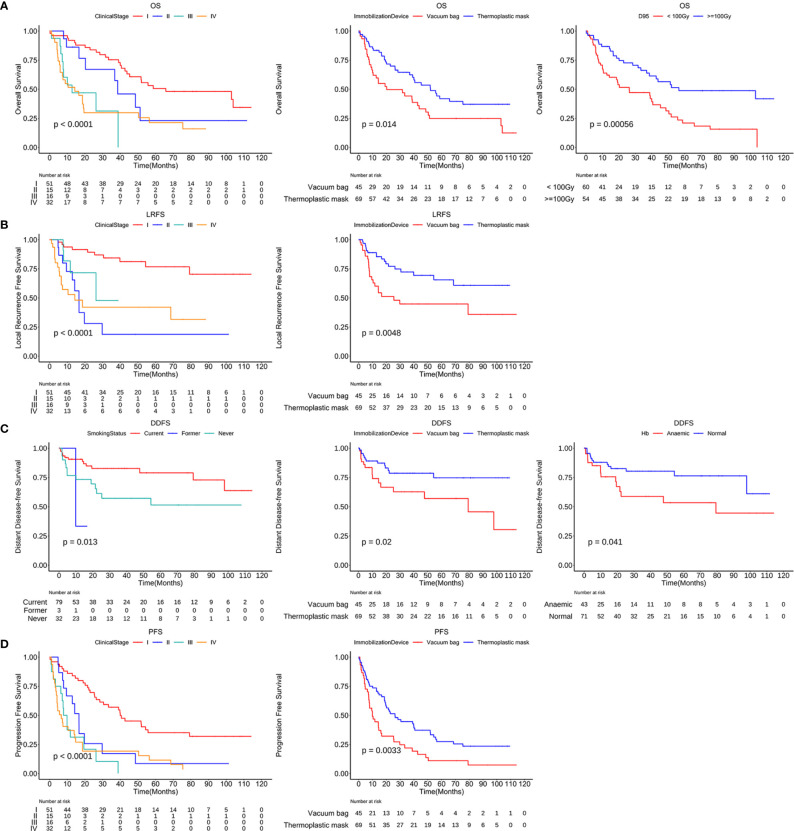
Kaplan-Meier curves of OS, LRFS, DDFS and PFS stratified by independent risk factors. OS curves stratified by the clinical stage, immobilization device and D95 **(A)**. LRFS curves **(B)** and PFS curves **(D)** stratified by the clinical stage and immobilization device. DDFS curves stratified by the smoking status, immobilization device and anemia or not **(C)**. D95, the prescription dose covers 95% of the target area; OS, overall survival; LRFS, local recurrence free survival; DDFS, distant disease-free survival; PFS, progression free survival.

**Figures 4** and **5** display the multivariable analysis for independent prognostic factors of long-term outcomes. As it can be seen, there were three independent prognostic factors, including clinical stage, immobilization device, and D95 that were associated with the prediction of OS in the multivariate analysis. In addition, the clinical stage and immobilization device were both statistically significant predictive factors of LRFS and PFS. The multivariate model showed that the smoking status, Hb and immobilization device were significant prognostic factors for DDFS. Patients with higher clinical stage tumors have three-fold increased risk of death (hazard ratio (HR), 3.282, 95%CI, 1.630-6.609, p<0.001) and tumor progression (HR, 3.476, 95%CI, 1.803-6.701, p<0.001), five-fold increased risk of local recurrence (HR, 5.060, 95%CI, 2.215-11.562, p<0.001), compared with lower clinical stage. Moreover, for patients fixed with thermoplastic masks, better OS (HR, 0.536, 95%CI, 0.300-0.958, p=0.035), LRFS (HR, 0.510, 95%CI, 0.265-0.982, p=0.044), DDFS (HR, 0.400, 95%CI, 0.181-0.886, p=0.024), and PFS (HR, 0.609, 95%CI, 0.384~0.967, p=0.036) rates could be observed compared to those with vacuum bags. Another important factor was D95, D95≧100Gy was significantly correlated with better OS (HR, 0.459, 95%CI, 0.245-0.862, p=0.015). Non-smokers (HR, 2.627, 95%CI, 1.107-6.233, p=0.029) and anemia (HR, 0.388, 95%CI, 0.169-0.892, p=0.026)were both associated with an increased risk of distant metastasis.

### The Prediction Nomogram Models Construction

**Figure 6:**
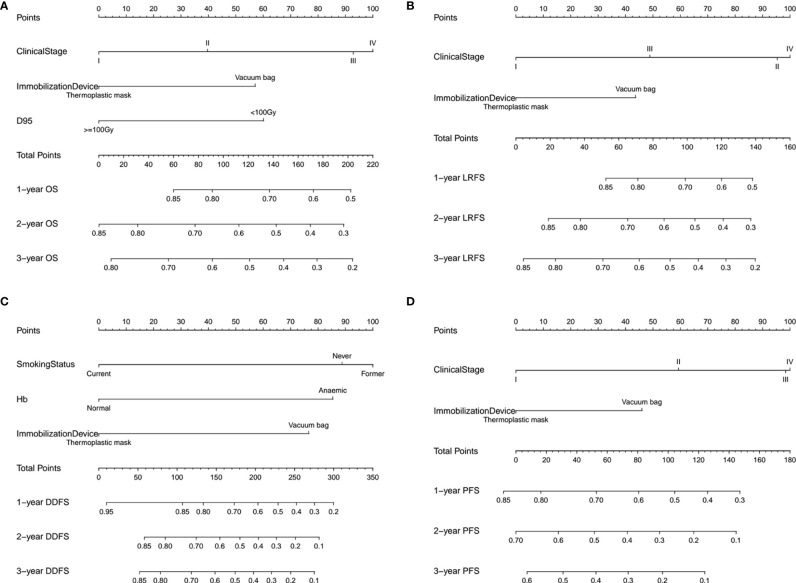
The nomogram containing identified factors for the 1-, 2-, and 3-year OS, LRFS, DDFS and PFS prediction of lung cancer patients. **(A)** Nomogram for OS, **(B)** nomogram for LRFS, **(C)** nomogram for DDFS and **(D)** nomogram for PFS. D95, the prescription dose covers 95% of the target area; OS, overall survival; LRFS, local recurrence free survival; DDFS, distant disease-free survival; PFS, progression free survival.

**Figure 7:**
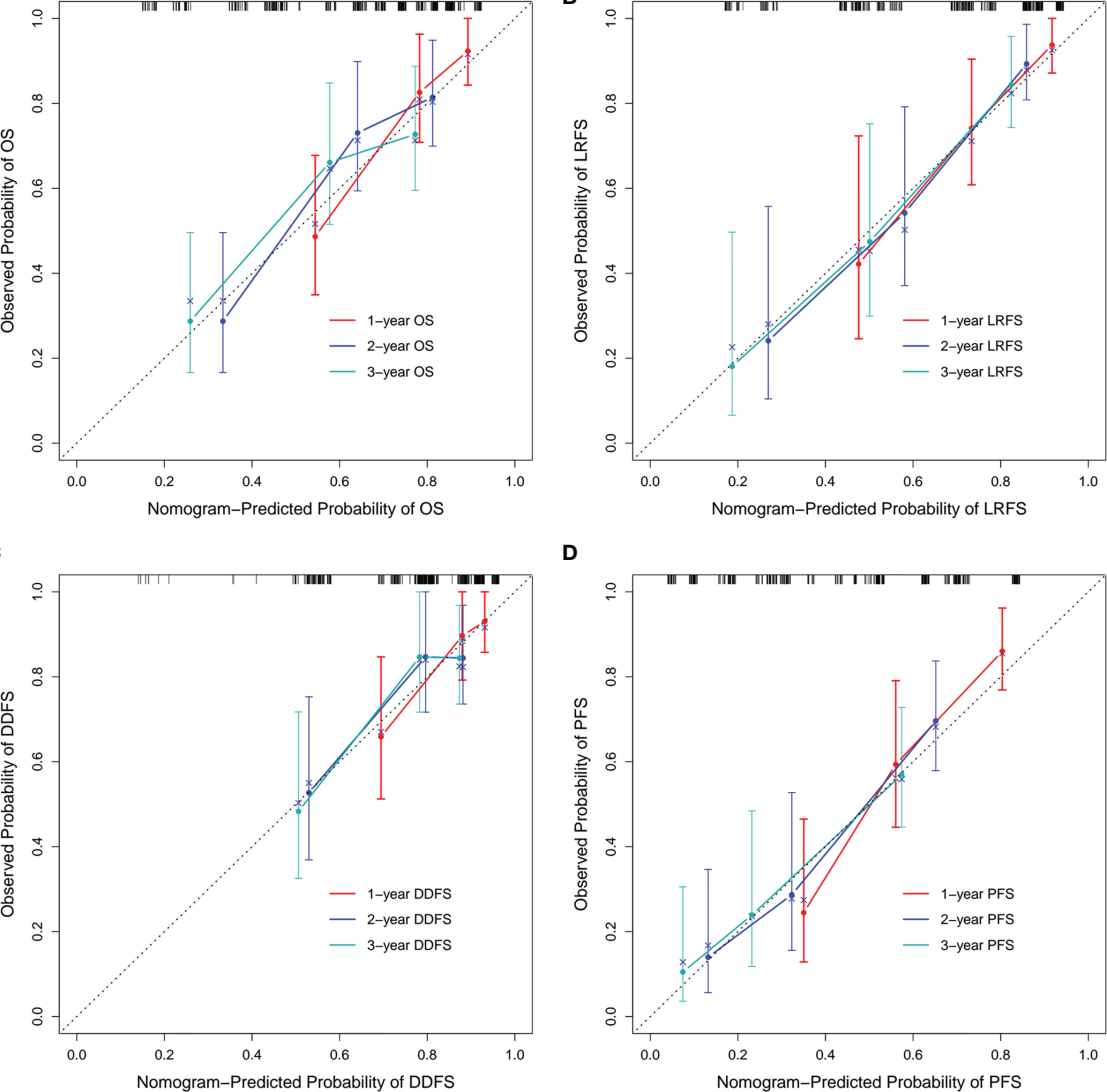
Calibration curves of each nomogram model. The calibration curves of the 1-, 2-, and 3-year OS prediction nomogram **(A)**, LRFS prediction nomogram **(B)**, DDFS prediction nomogram **(C)** and PFS prediction nomogram **(D)**. OS, overall survival; LRFS, local recurrence free survival; DDFS, distant disease-free survival; PFS, progression free survival.

In addition, we built four prediction nomogram models to predict 1-year, 2-year, and 3-year OS, LRFS, DDFS, and PFS through integrating corresponding significant factors identified *via* multivariate analysis (**Figure 6**). The C-index of the nomogram models for OS, LRFS, DDFS, and PFS were 0.705 (95% CI 0.634–0.777), 0.763 (95% CI 0.690–0.835), 0.688 (95% CI 0.588–0.788), 0.701 (95% CI 0.645–0.757), respectively. The bootstrap-corrected calibration curves showed that the nomogram models for OS, LRFS, DDFS, and PFS all had good prediction efficiency (**Figure 7**). These results demonstrated that all the four prediction nomogram models might be prognostic tools used for the clinical management of lung cancer patients.

### Toxicities

As observed, early toxicities (i.e. grade 3 acute radiation pneumonitis) occurred in five patients (4.4%), and overtime late toxicities occurred in 24 patients (21.1%) after treatment. Radiation pneumonitis and bone complications were the most concerning late toxicities in our cohort. Grade 1 and 2 late radiation pneumonitis occurred in 10 (8.8%) and seven patients (6.1%), respectively. Grade 1, 2, and 4 late bone complications occurred in 1 (1.8%), 1 (0.9%), and 3 patients (2.6%), respectively, without grade 3 or 5 toxicities. Furthermore, we found that one patient had both grade 1 radiation pneumonitis and grade 2 late bone complications. No treatment-related fatal events (i.e. grade 5) were seen in our patient group.

## Discussion

In this study, we report outstanding OS, LRFS, DDFS, PFS and toxicity of inoperable lung cancer treated with SBRT. Our results confirm that some clinical and dosimetric factors, such as clinical stage, Hb, immobilization device, and D95 correlated with the clinical outcomes. We have established nomogram models based on identified factors, which could predict individual clinical outcomes with satisfactory accuracy. This method is helpful in stratifying patients into low or high-risk groups, optimizing SBRT treatment plans, and guiding follow-up management and strategies. SBRT was the major treatment of choice for medically inoperable patients with primary lung cancer due to its therapeutic effectiveness and the prognosis rival surgical prognosis in operable patients, and previously reported low rates of toxicity and side effects (2, 3). Baumann et al. (17) presented results of a formal phase II clinical trial using SBRT regimen in NSCLC patients, with a 1-year, 2-year, 3-year OS of 86%, 65%, 60%, and a 3-year LRFS and PFS of 92% and 52%. Our literature review also confirmed that other studies described similar clinical outcomes (18). Our results were comparable to those reports: the OS for lung SBRT at 1-, 2-, 3- and 5- years were 74.8%, 61.7%, 56.3% and 34.7%, respectively, with LRFS, DDFS, PFS still acceptable at 1-, 2-, 3- and 5-year. We have also concluded that toxicity was an endpoint of concern, so our results are consistent with other reported studies. We verified a rate of 7% of grade 3 or higher toxicities, indicating that the safety of SBRT is reassuring (19).

Our data, along with the results explained in other similar reports, have demonstrated that the clinical stage has been associated with OS, LRFS, and PFS. We found that patients with lower clinical stage had a better OS, LRFS and PFS than that of patients with more severe clinical stage (20, 21). Some clinical trials indicate that larger tumors require higher doses (22). In order to lower treatment-related toxicity, we did not change the dose and fraction schedule according to the tumor size, and this may be the reason for the decrease of OS, LRFS, and PFS in patients with more severe clinical stages. Another possibility is that lung CT would underestimate the tumor size under the microscope. However, SBRT does not point at the microscopic extension (23). Therefore, the clinical stage was taken as an independent prognostic factor for OS, LRFS, and PFS, and should be considered in making treatment decisions.

Device accuracy provides high dose radiation to the target volume while avoiding harming organs at risk. Thus, it can be said that an accurate, reproducible, and appropriate immobilization device is essential for lung cancer patients treated with SBRT (24, 25). In addition, whether the type of immobilization system has a significant impact on long-term outcomes in patients with this disease is worth studying and paying further attention. We found that patients fixed with thermoplastic masks had significantly better results than those with vacuum bags and found other publications that reinforced our conclusions. For instance, Navarro-Martin et al. (26) observed that thermoplastic masks have better reproducibility and significantly reduce the effect of respiration, tumor displacement, and set-up errors compared to vacuum bags. Our results also confirm that thermoplastic masks may be better than vacuum bags for the localization system used during SBRT treatments of lung cancer patients.

In the multivariate analyses of dosimetric factors, only D95 was highly correlated. Several published studies have already shown that BED≧100Gy is significantly correlated with better OS (27), and it is in line with practical guidelines (4, 28). Our findings concurred with Guckenberger’ results that proved that D95≧100Gy was most significantly correlated with a better probability of OS. Therefore, on the premise of no contraindications, the prescription dose in BED of lung SBRT should be ≧100Gy.

In some cohort studies for lung cancer patients, the presence of anemia was considered a significant independent factor for worse outcomes (29, 30). Our result is consistent with historical reports that anemia is an independent risk factor associated with poor DDFS in univariate analysis and multivariate analyses. Diminished oxygen availability or hypoxia can increase tumor aggressiveness and promote cancer recurrence and metastasis *via* several potential mechanisms, such as the production of growth factors and reduction of apoptotic potential (31, 32). On the basis of our results, we recommend that diagnosis and correction of anemia should be considered before and during treatment. As previously demonstrated by Underner M. et al. (33), smoking was an independent risk factor related to adverse outcomes. On the contrary, we found that smokers are less likely to have distant metastasis. This may be due to the short follow-up time of some cases, which limits the capture of distant metastasis of smokers.

Other scholars in the field have previously studied the association between clinical and dosimetric factors and its clinical outcomes. However, multiple factors are rarely combined to evaluate prognosis. Nomograms can integrate multiple factors into a mathematical model that graphically shows the probability of clinical outcomes (34). What’s more, nomograms were considered more accurate and effective tools in predicting the prognosis (35). In our study, four prediction nomogram models were built based on identified clinical and dosimetric factors to effectively predict the probability of 1-, 2-, and 3-year clinical outcomes in different lung cancer patients. Similarly, the calibration curves showed satisfactory consistency between the nomogram models and the practical observation results. This prognostic information of nomograms can guide physicians to make accurate clinical decisions for individual patients, including appropriate SBRT regimens planning and future systematic treatment strategy, which is helpful in prolonging patients’ survival and improving their quality of life. Considering the small sample issues, we did not divide patients into training and validation groups, which is why the internal verification method was adopted in our study.

Some of the limitations of our study include its retrospective nature and heterogeneity of fraction schedules. Our results should be further examined in a larger prospective randomized comparative clinical trial. Additionally, some lung cancer patients did not have pathological confirmation of pulmonary lesions. However, SBRT may be a safe and practical choice for pulmonary lesions without pathological warranty, and its therapeutic effect is almost the same as that of patients suffering from non-small cell lung cancer (36).

## Conclusions

In conclusion, we successfully demonstrated that SBRT for lung cancer patients achieves good OS, LRFS, DDFS, and PFS, while maintaining acceptable severe toxicity rates. Furthermore, our data also indicates that the clinical stage, Hb, immobilization device, and D95 are independent prognostic factors for the clinical outcomes. Moreover, the nomograms we proposed are good for 1-, 2-, and 3-year OS, LRFS, DDFS, PFS prediction for lung cancer patients undergoing SBRT.

## Data Availability Statement

The raw data supporting the conclusions of this article will be made available by the authors, without undue reservation.

## Author Contributions

The author’s responsibilities were as follows: B-TH and L-ML conceived and designed the study. C-HS, L-ML, and YW contributed to data collection. L-ML, YW, and P-XL analyzed data and interpreted data. L-ML and B-TH wrote paper. B-TH had primary responsibility for final content. All authors contributed to the manuscript review and approved the final version.

## Funding

This work was sponsored by National Natural Science Foundation of China (Grant numbers 81602667), Medical Scientific Research Foundation of Guangdong Province (Grant numbers A2015534).

## Conflict of Interest

The authors declare that the research was conducted in the absence of any commercial or financial relationships that could be construed as a potential conflict of interest.

## Publisher’s Note

All claims expressed in this article are solely those of the authors and do not necessarily represent those of their affiliated organizations, or those of the publisher, the editors and the reviewers. Any product that may be evaluated in this article, or claim that may be made by its manufacturer, is not guaranteed or endorsed by the publisher.

## Abbreviations

SBRT, Stereotactic body radiation therapy; OS, overall survival; LRFS, local recurrence free survival; DDFS, distant disease-free survival; PFS, progression free survival; 4DCT, four-dimensional computed tomography; 3DCT, three-dimensional computed tomography; ITV4D, internal target volume based on 4DCT; ITV3D, internal target volume based on 3DCT; GTV, gross tumor volumes; PTV, planning target volume; CBCT, cone beam computed tomography; KPS, Karnofsky performance status; BMI, body mass index; Hb, hemoglobin; NLR, Neutrophil-to-Lymphocyte ratio; PLR, Platelet-to-Lymphocyte ratio; D95, prescription dose covers 95% of the target area; Dmax, the maximum dose in the whole plan; PTVmin, the minimum dose of PTV; PTVmean, mean dose of PTV; PTVmax, the maximum dose of PTV; PTVmin/PTVmax, dose inhomogeneity of PTV; GTVmin, the minimum dose of GTV; GTVmean, mean dose of GTV; GTVmax, the maximum dose of GTV; GTVmin/GTVmax, dose inhomogeneity of GTV; BED, biologically effective doses; VIF, variance inflation factor; C-index, concordance index; 95%CI, 95% confidence interval; HR, hazard ratio.
